# Prognostic Influence of Lung Compliance in Patients with Cardiogenic Shock and Invasive Mechanical Ventilation

**DOI:** 10.31083/j.rcm2511420

**Published:** 2024-11-22

**Authors:** Jonas Rusnak, Tobias Schupp, Kathrin Weidner, Marinela Ruka, Sascha Egner-Walter, Alexander Schmitt, Muharrem Akin, Péter Tajti, Kambis Mashayekhi, Mohamed Ayoub, Michael Behnes, Ibrahim Akin

**Affiliations:** ^1^Department of Cardiology, Angiology, Haemostaseology and Medical Intensive Care, University Medical Centre Mannheim, Medical Faculty Mannheim, Heidelberg University, 68167 Mannheim, Germany; ^2^Department of Cardiology and Angiology, Hannover Medical School, 30625 Hannover, Germany; ^3^Gottsegen György National Cardiovascular Center, 1096 Budapest, Hungary; ^4^Department of Internal Medicine and Cardiology, Mediclin Heart Centre Lahr, 77933 Lahr, Germany; ^5^Division of Cardiology and Angiology, Heart Center University of Bochum, 32545 Bad Oeynhausen, Germany

**Keywords:** cardiogenic shock, mechanical ventilation, lung compliance, prognosis, mortality

## Abstract

**Background:**

There is limited data regarding the influence of lung compliance on the outcome of patients with cardiogenic shock (CS). Thus, a registry study was conducted to assess the prognostic influence of lung compliance in invasively ventilated patients with CS.

**Methods:**

Hospital records for consecutive invasively ventilated CS-patients from June 2019 to May 2021 were collected into a prospective registry. Our study evaluated the prognostic influence of lung compliance on 30-day all-cause mortality. Statistical analyses comprised *t*-tests, analysis of variance (ANOVA), Kruskal-Wallis-tests, Spearman’s correlation, Kaplan-Meier survival analyses, and Cox regression.

**Results:**

A total of 141 patients with CS requiring invasive mechanical ventilation were included. Stratification by quartiles revealed that patients with the lowest lung compliance (≤23.8 mL/cmH_2_O) experienced the highest mortality rates (77.1% vs. 66.7% vs. 48.6% vs. 51.4%; log-rank *p* = 0.018) both overall and among the subgroup of CS-patients with cardiac arrest (80% vs. 74% vs. 53% vs. 59%; log-rank *p* = 0.037). After stratifying by the median, patients with lung compliance <30.4 mL/cmH_2_O demonstrated a significantly higher 30-day all-cause mortality compared to those above this threshold (71.8% vs. 50.0%; log-rank *p* = 0.007) for both the overall cohort and the cardiac arrest subgroup (77.2% vs. 55.9%; log-rank *p* = 0.008). Multivariable adjustment confirmed that lung compliance <30.4 mL/cmH_2_O was significantly associated with increased 30-day all-cause mortality in the entire cohort (hazard ratio [HR] = 1.698; 95% CI 1.085–2.659; *p* = 0.021). Notably, this association was not significant in CS-patients with cardiac arrest (HR = 1.523; 95% CI 0.952–2.438; *p* = 0.080). Additionally, those with lung compliance below the median experienced fewer ventilator-free days (*p* = 0.003).

**Conclusions:**

In invasively ventilated CS-patients, low lung compliance was associated with higher all-cause mortality and fewer ventilator-free days at 30 days.

**Clinical Trial Registration:**

NCT05575856, https://clinicaltrials.gov/study/NCT05575856.

## 1. Introduction

Mortality in patients with cardiogenic shock (CS) remains high, particularly when complicated by respiratory failure [1, 2]. Strategies for treating patients with CS derives from research conducted on critically ill individuals and those affected by acute respiratory distress syndrome (ARDS) [2, 3]. Lung compliance, the elasticity or stretchability of the lungs, is a known ARDS risk factor associated with poorer outcome [4].

In patients with cardiovascular issues, such as those suffering from heart failure, a decrease in lung compliance occurs regardless of left ventricular failure classification, as defined by the New York Heart Association (NYHA) class or pulmonary vascular pressure [5, 6, 7]. Additionally, patients with cardiopulmonary edema often present with reduced lung compliance [8]. Previous studies, however, focused only on spontaneously breathing individuals, and estimating compliance through measurements of total lung capacity and esophageal pressure [5, 6, 7, 8].

A key hinderance in the field is that limited data exists on lung compliance in CS patients who require invasive mechanical ventilation. A sub analysis of the LUNG SAFE study, which included patients with isolated cardiopulmonary edema, demonstrated that higher peak-, plateau- and driving-pressure were associated with increased hospital mortality, correlating with decreased lung compliance [9]. Thus, lung compliance may serve as a critical prognostic marker for adverse outcomes in ventilated cardiac patients. Furthermore, several studies have shown that higher lung compliance in patients with cardiac arrest correlates with improved short- and long-term survival, as well as better neurological outcome [10, 11, 12]. The present study aims to further elucidate the prognostic significance of lung compliance in patients with CS undergoing invasive mechanical ventilation.

## 2. Materials and Methods

### 2.1 Study Design, Inclusion and Exclusion Criteria

This analysis is based on the data of the “Cardiogenic Shock Registry Mannheim” (CARESMA-registry). The CARESMA-registry represents a prospective single-center registry enrolling consecutive CS-patients admitted to the internal intensive care unit (ICU) of the University Medical Center Mannheim, Germany (clinicaltrials.gov identifier: NCT05575856) from June 2019 to May 2021, as recently published [13]. The registry did not include patients who passed away prior to admission to the ICU or catheterization laboratory. The registry, which was created in compliance with the principles of the Declaration of Helsinki, was approved by the medical ethics committee II of the Medical Faculty Mannheim, University of Heidelberg, Germany. Mannheim’s Medical Ethics Committee II waived the requirement for study-specific informed consent.

**Fig. 1.**
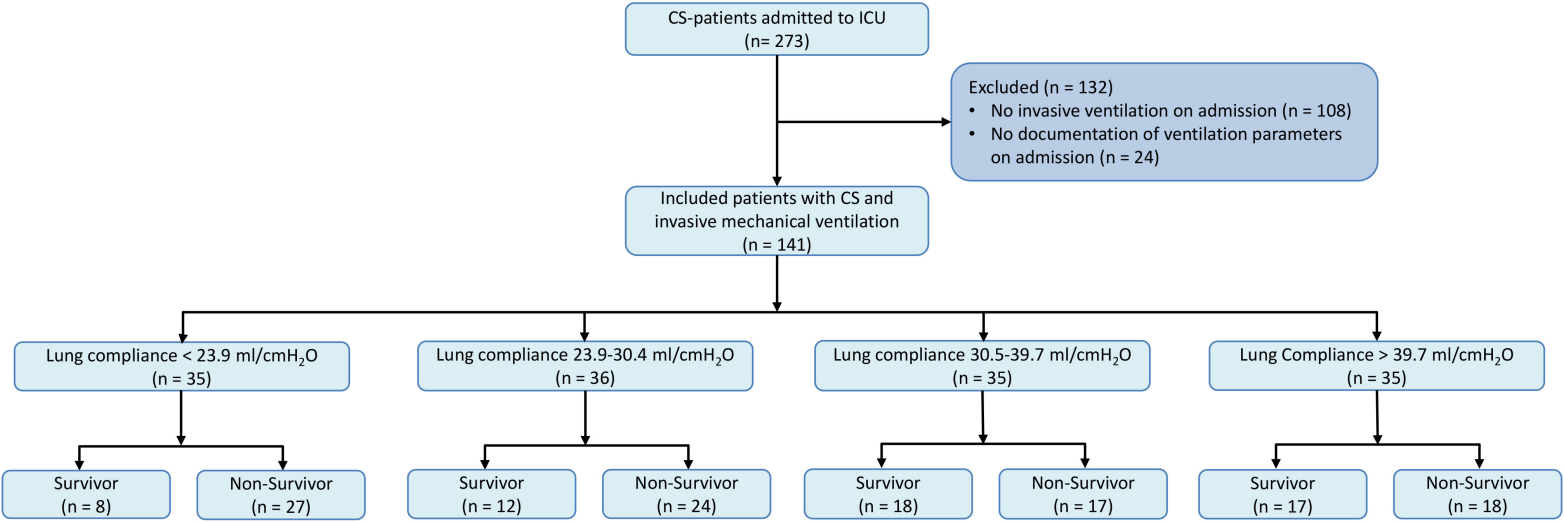
**Patient selection for analysis of lung compliance in CS patients requiring mechanical ventilation**. Fig. 1 illustrates the stepwise selection process used to identify the study cohort. Initially, 108 patients not receiving invasive mechanical ventilation were excluded. Further, 24 patients lacking comprehensive ventilatory parameters at admission were also excluded. This flowchart delineates the exclusion criteria applied and the final cohort included for analysis. CS, cardiogenic shock; ICU, intensive care unit.

In this analysis, we focused exclusively on patients with CS-patients who required invasive mechanical ventilation. Initially, all patients from the CARESMA registry not undergoing invasive mechanical ventilation were excluded totaling 108 individuals. Subsequently, patients were excluded due to inadequate documentation of ventilatory parameters on admission (n = 24). No additional exclusion criteria were applied in this study. A flow-chart of the inclusion and exclusion process is given in Fig. 1.

### 2.2 Data Collection

Data, such as consult notes, laboratory and vital parameter, admission paperwork, and treatment data, were organized using the electronic hospital database and the patient data management system (ICCA, Philips, Philips GmbH Market DACH, Hamburg, Germany). Ventilatory parameters were recorded using the patient data management system, and the attached respiratory machines data. Due to the pressure-controlled ventilation used in our ICU, peak inspiratory pressure (PIP) was used instead of plateau pressure. Positive end expiratory pressure (PEEP) was subtracted from PIP to determine the dynamic driving pressure.

### 2.3 Definition of Cardiogenic Shock and Lung Compliance

CS was defined by persisting (>30 min) hypotension (systolic blood pressure less than 90 mmHg) or need for vasopressor or inotropic therapy to achieve systolic blood pressure higher than 90 mmHg [14]. In addition, indicators of end-organ hypoperfusion, such as oliguria (urine production <30 mL/h), altered mental state, clammy, cold skin, or elevated lactate (>2 mmol/L) must have been present. Moreover, patterns (increased left ventricular end diastolic pressure >20 mmHg, elevated pulmonary capillary wedge pressure diagnosed by pulmonary artery catheterization or by mitral E-wave deceleration time ≤130 ms in echocardiography) or indirect signs (pulmonary congestion confirmed by clinical examination or chest X-ray) of elevated left ventricular filling pressures were mandatory for CS diagnosis. To finally confirm that CS was the fundamental cause of critical illness, a distinct cause of CS was required. Since cardiac tamponade and pulmonary embolism are defined as probable underlying causes of acute heart failure and CS in current guidelines and recommendations, they were identified as forms of CS despite the obstructive mode of shock [15, 16, 17, 18]. When aortic valve regurgitation was the primary source of hemodynamic instability, aortic dissection was classified as CS [16]. Both ventricular tachycardia, such as intrinsic ventricular fibrillation, and supraventricular arrhythmias, such as atrial fibrillation, flutter, or tachycardia, were potential arrhythmic causes of CS.

Lung compliance is a measure for the expansion capability of the lung tissue. It is defined by the volume (tidal volume) that can be administered by the given change in pressure (driving pressure). Lung compliance was automatically recorded on admission by respiratory machines. If lung compliance was not documented by the connected ventilators, lung compliance was calculated using the first measured dynamic driving pressure and tidal volume (tidal volume/dynamic driving pressure).

### 2.4 Study Endpoints

The primary endpoint was defined as mortality at 30 days post-enrollment. All-cause mortality data were obtained using the electronic hospital database and direct communication with state resident registration offices (“bureau of mortality statistics”). Verification of patient identities was ensured through recorded data including the day of birth, residential address, place of birth, and surname. Throughout the 30-day follow-up period, no patients were lost. Furthermore, as a secondary endpoint, ventilator-free days were evaluated. The method for calculating ventilator-free days followed established guidelines [19]: (1) patients who died within the follow-up of 30 days received zero ventilator-free days; (2) patients successfully extubated *x* days post-ventilation initiation were credited with 30 – x ventilator-free days; (3) patients requiring ventilation for days or more were assigned zero ventilator-free days; (4) the day of intubation was defined as day 0; (5) an extubation deemed successful was defined as lasting over 48 hours without the need for re-intubation.

### 2.5 Statistical Methods

Quantitative data is shown as median and interquartile range (IQR) or mean and standard error of the mean (SEM), as appropriate. To find deviations from the Gaussian distribution, the Kolmogorov-Smirnov test was used. Thereafter, depending on the compared groups the Kruskal-Wallis-test, analysis of variance (ANOVA) test, Student’s *t* test or Mann-Whitney U test were performed to compare the means of the groups. Chi-square or Fisher’s exact test were used to compare qualitative data, which are given as absolute and relative frequencies. Spearman’s correlation was used to correlate lung compliance with clinical and laboratory parameters. Kaplan-Meier analyses on 30-day survival according to the quartiles of lung compliance (lung compliance <23.9 mL/cmH_2_O vs. lung compliance 23.9–30.4 mL/cmH_2_O vs. lung compliance 30.5–39.7 mL/cmH_2_O vs. lung compliance >39.7 mL/cmH_2_O) were performed in the entire cohort as well as the subgroup of patients with cardiac arrest. Furthermore, Kaplan-Meier analysis with the differentiation for the median of lung compliance were executed (lung compliance <30.4 mL/cmH_2_O vs. lung compliance ≥30.4 mL/cmH_2_O). Using Cox regression, univariate hazard ratios (HR) and 95% confidence intervals (CI) were obtained. Care was taken to solely consider clinically relevant variables for inclusion in Cox regression analyses such as age, body mass index, sex, lactate, norepinephrine, troponin I, acute physiology score, SCAI (Society of Cardiovascular Angiography & Interventions) shock stage, PaCO_2_ (partial arterial pressure of carbon dioxide), PaO_2_/FiO_2_ (partial arterial pressure of oxygen/fraction of inspired oxygen), pneumonia, PEEP, peak inspiratory pressure, cause of CS, and troponine I levels. Thereafter, multivariable regression models were developed using the “Backwards selection method” including variables with *p*-values < 0.10 in the univariable analyses. The final multivariable Cox regression model includes solely variables that were selected after the selection process. The entry *p*-value for the multivariable models was *p* < 0.10 and the stay *p*-value was *p* < 0.05.

All statistical test results were rated significant when the *p*-value was <0.05. Statistical analyses were performed with SPSS (Version 29, IBM, Armonk, NY, USA).

## 3. Results

Table 1 outlines the baseline characteristics stratified by quartiles of lung compliance. The groups showed comparable baseline characteristics considering age, body mass index and prior medical history on admission. Patients in the lowest lung compliance quartile demonstrated a higher prevalence of smoking compared to those in higher quartiles (54.3% vs. 36.1% vs. 31.4% vs. 20.0%; *p* = 0.025). Additionally, these patients exhibited lower systolic blood pressures, escalating progressively across the quartiles to 102 mmHg, 109 mmHg, and 117 mmHg, respectively (*p* = 0.01). Despite the prevalence of lower systolic blood pressures, most patients maintained pressures above 90 mmHg, facilitated by the common use of vasopressors and dobutamine on admission. Patients in the lower quartiles were less often male compared to those in the higher quartiles (51.4% vs. 41.7% vs. 77.1% vs. 80.0%; *p* = 0.001). Furthermore, patients in the higher lung compliance quartiles had a lower prevalence of chronic heart failure (37.1% vs. 38.9% vs. 25.7% vs. 11.4%; *p* = 0.039) and were less often treated with diuretics (48.6% vs. 50.0% vs. 37.1% vs. 14.3%; *p* = 0.036).

**Table 1.** **Baseline demographic and clinical characteristics of patients stratified by lung compliance quartiles**.

			Lung compliance		Lung compliance		Lung compliance		Lung compliance		*p*-value
			<23.9 mL/cmH_2_O		23.9–30.04 mL/cmH_2_O		30.5–39.7 mL/cmH_2_O		>39.7 mL/cmH_2_O		
			(n = 35)		(n = 36)		(n = 35)		(n = 35)		
Age (median, IQR)			67	(60–77)	72	(61–82)	63	(56–77)	72	(59–78)	0.464
Male sex, n (%)			18	(51.4)	15	(41.7)	27	(77.1)	28	(80.0)	**0.001**
Body mass index, kg/m^2^ (median, IQR)			27.6	(24.5–30.5)	27.8	(25.6–31.2)	26.2	(24.2–30.9)	26.6	(24.5–29.2)	0.536
Clinical parameters (median, IQR)											
	Body temperature (°C)		35.6	(34.2–36.3)	35.8	(34.4–36.5)	34.9	(33.7–36.5)	35.2	(34.5–36.1)	0.562
	Heart rate (bpm)		93	(78–110)	95	(76–113)	85	(70–110)	88	(73–107)	0.770
	Systolic blood pressure (mmHg)		98	(86–116)	102	(84–125)	109	(96–131)	117	(103–139)	**0.010**
	Respiratory rate (breaths/min)		19	(16–23)	20	(18–23)	18	(15–20)	19	(17–22)	0.050
Cardiovascular risk factors, n (%)											
	Arterial hypertension		22	(62.9)	26	(72.2)	21	(60.0)	26	(74.3)	0.506
	Diabetes mellitus		16	(45.7)	10	(27.8)	12	(34.3)	7	(20.0)	0.126
	Hyperlipidemia		15	(42.9)	17	(47.2)	16	(45.7)	19	(54.3)	0.803
	Smoking		19	(54.3)	13	(36.1)	11	(31.4)	7	(20.0)	**0.025**
Prior medical history, n (%)											
	Coronary artery disease:		11	(31.4)	11	(30.6)	12	(34.3)	8	(22.9)	0.754
		1–vessel disease	2	(5.7)	5	(13.9)	4	(11.4)	3	(8.6)	
		2–vessel disease	3	(8.6)	1	(2.8)	0	(0.0)	0	(0.0)	0.456
		3–vessel disease	6	(17.1)	5	(13.9)	8	(22.9)	5	(14.3)	
	Chronic heart failure		13	(37.1)	14	(38.9)	9	(25.7)	4	(11.4)	**0.039**
	Atrial fibrillation		13	(37.1)	11	(30.6)	6	(17.1)	7	(20.0)	0.195
	Chronic kidney disease		12	(34.3)	13	(36.1)	5	(14.3)	6	(17.1)	0.067
	Stroke		8	(22.9)	3	(8.3)	5	(14.3)	3	(88.6)	0.241
	COPD		13	(37.1)	8	(22.2)	8	(20.0)	4	(11.4)	0.077
	Liver cirrhosis		1	(2.9)	2	(5.6)	0	(0.0)	2	(5.7)	0.542
Medication on admission, n (%)											
	ACE-inhibitor		11	(31.4)	8	(22.2)	9	(25.7)	9	(25.7)	0.850
	ARB		6	(17.1)	6	(16.7)	6	(17.1)	6	(17.1)	1.000
	Beta-blocker		18	(51.4)	19	(52.8)	13	(37.1)	9	(25.7)	0.067
	ARNI		1	(2.9)	1	(2.9)	0	(0.0)	1	(2.9)	0.803
	Mineralocorticoid antagonist		5	(14.3)	5	(13.9)	4	(11.4)	5	(14.3)	0.982
	SGLT-2 inhibitor		2	(5.7)	0	(0.0)	2	(5.9)	2	(5.7)	0.550
	Diuretics		17	(48.6)	18	(50.0)	13	(37.1)	5	(14.3)	**0.036**
	ASA		7	(20.0)	10	(27.8)	4	(11.4)	11	(31.4)	0.193
	P2Y12-inhibitor		5	(14.3)	0	(0.0)	4	(11.4)	1	(2.9)	0.059
	Statin		13	(37.1)	16	(44.4)	12	(34.3)	11	(31.4)	0.698

ACE, angiotensin-converting-enzyme; ARB, angiotensin receptor blocker; ARNI, angiotensin receptor neprilysin inhibitor; ASA, acetylsalicylic acid; bpm, beats per minute; COPD, chronic obstructive pulmonary disease; IQR, interquartile range; SGLT-2, sodium-glucose co-transporter-2. Level of significance *p* < 0.05. Bold type indicates statistical significance.

As presented in Table 2, causes of CS and the distribution within the SCAI shock classification were consistent across all groups. Predominantly, patients in this study presented with advanced stages of CS, which was reflected by the significant need for cardiopulmonary resuscitation in 82.3% of cases. This high incidence of SCAI shock stage E was primarily attributed to the frequency of cardiac arrest at admission rather than their hemodynamic condition alone. Regarding lung compliance, the group with values greater than 39.7 mL/cmH_2_O exhibited the lowest prevalence of left ventricular ejection fraction <30% (60.0% vs. 63.9% vs. 60.0% vs. 25.7%; *p* = 0.001) and the smallest average diameter of the inferior vena cava (1.8 cm vs. 2.1 cm vs. 2.0 cm vs. 1.7 cm; *p* = 0.024). Conversely, patients in the lower quartiles of lung compliance had reduced incidences of shockable rhythms (31.4% vs. 27.8% vs. 60.0% vs. 51.4%; *p* = 0.032) and higher occurrences of non-shockable rhythms (54.3% vs. 47.2% vs. 28.6% vs. 25.7%; *p* = 0.032) compared to those in the higher quartiles.

**Table 2.** **Cardiogenic shock metrics, follow-up, and endpoints stratified by lung compliance quartiles**.

			Lung compliance		Lung compliance		Lung compliance		Lung compliance		*p*-value
			<23.9 mL/cmH_2_O		23.9–30.4 mL/cmH_2_O		30.5–39.7 cmH_2_O		>39.7 mL/cmH_2_O		
			(n = 35)		(n = 36)		(n = 35)		(n = 35)		
Etiology of CS, n (%)											
	Acute myocardial infarction		15	(42.9)	15	(41.7)	23	(65.7)	18	(51.4)	0.128
	Arrhythmia		2	(5.7)	2	(5.6)	5	(14.3)	5	(14.3)	
	Acute decompensated heart failure		13	(37.1)	12	(33.3)	2	(5.7)	7	(20.0)	
	Valvular heart disease		1	(2.9)	2	(5.6)	0	(0.0)	1	(2.9)	
	Cardiomyopathy		1	(2.9)	1	(2.8)	3	(8.6)	0	(0.0)	
	Pulmonary embolism		3	(8.6)	4	(11.1)	1	(2.9)	4	(11.4)	
	Pericardial temponade		0	(0.0)	0	(0.0)	1	(2.9)	0	(0.0)	
SCAI classification of CS, n (%)											
	Stage C		5	(14.3)	7	(19.4)	2	(5.7)	6	(17.1)	0.469
	Stage D		0	(0.0)	2	(5.6)	1	(2.9)	2	(5.7)	
	Stage E		30	(85.7)	27	(75.0)	32	(91.4)	27	(77.1)	
Transthoracic echocardiography											
	LVEF >55%, n (%)		3	(8.6)	5	(13.9)	2	(5.7)	3	(8.6)	**0.001**
	LVEF 54–41%, n (%)		1	(2.9)	1	(2.8)	2	(5.7)	11	(31.4)	
	LVEF 40–30%, n (%)		7	(20.0)	7	(19.4)	8	(22.9)	11	(31.4)	
	LVEF <30%, n (%)		21	(60.0)	23	(63.9)	21	(60.0)	9	(25.7)	
	LVEF not documented, n (%)		3	(8.6)	0	(0.0)	2	(5.7)	1	(2.9)	
	Inferior vena cava, cm (median, IQR)		1.8	(1.6–1.9)	2.1	(1.9–2.4)	2.0	(1.8–2.2)	1.7	(1.5–2.0)	**0.024**
	TAPSE, mm (median, IQR)		14	(12–23)	15	(13–21)	14	(9–20)	18	(17–24)	0.274
Cardiopulmonary resuscitation											
	Out-of-hospital cardiac arrest, n (%)		20	(57.1)	19	(52.8)	24	(68.6)	25	(71.4)	0.126
	In-hospital cardiac arrest, n (%)		10	(28.6)	8	(22.2)	8	(22.9)	2	(5.7)	
	Shockable rhythm, n (%)		11	(31.4)	10	(27.8)	21	(60.0)	18	(51.4)	**0.032**
	Non-shockable rhythm, n (%)		19	(54.3)	17	(47.2)	10	(28.6)	9	(25.7)	
	ROSC, min (mean, SEM)		31	(±6)	18	(±3)	22	(±5)	19	(±4)	0.225
Respiratory status											
	Duration of mechanical ventilation, days, (mean, SEM)		8	(±2)	7	(±1)	8	(±1)	6	(±1)	0.642
	PaO_2_/FiO_2_ ratio, (mean, SEM)		213	(±27)	207	(±30)	278	(±27)	251	(±30)	0.263
	PaCO_2_, mmHg (mean, SEM)		52	(±3)	45	(±2)	43	(±2)	43	(±2)	**0.045**
	PEEP, cmH_2_O (mean, SEM)		8	(±0.4)	9	(±0.5)	7	(±0.4)	7	(±0.4)	**0.003**
	Peak inspiratory pressure, cmH_2_O (median, IQR)		31	(26–34)	25	(22–29)	20	(18–22)	17	(15–20)	**0.001**
	Dynamic driving pressure, cmH_2_O (median, IQR)		19	(17–26)	16	(15–19)	13	(11–14)	11	(9–12)	**0.001**
	Tidal volume, ml (mean, SEM)		371	(±18)	451	(±12)	458	(±13)	580	(±29)	**0.001**
Multiple organ support during ICU											
	Vasopressor treatment, n (%)		31	(88.6)	34	(94.4)	29	(82.9)	32	(91.4)	0.438
	Inotropic treatment, n (%)		15	(42.9)	10	(27.8)	9	(25.7)	11	(31.4)	0.418
	Norepinephrine dose on admission, µg/kg/min (median, IQR)		0.2	(0.1–1.1)	0.2	(0.1–0.4)	0.1	(0.1–0.5)	0.2	(0.1–0.3)	0.559
	Dobutamine, cumulative dose day 1, mg/kg (median, IQR)		4	(2–21)	4	(1–10)	11	(5–20)	3	(2-15)	0.289
	Extracorporeal life support, n (%)		7	(20.0)	5	(13.9)	10	(28.6)	1	(2.9)	0.153
		Micro-axial flow pump, n (%)	3	(8.6)	0	(0.0)	0	(0.0)	1	(2.9)	0.100
		Veno-arterial ECMO, n (%)	6	(17.1)	5	(13.9)	10	(28.6)	0	(0.0)	**0.009**
	Renal replacement therapy, n (%)		15	(42.9)	9	(25.7)	13	(37.1)	14	(41.2)	0.455
Baseline laboratory values											
	pH (median, IQR)		7.21	(7.15–7.28)	7.25	(7.19–7.31)	7.27	(7.19–7.32)	7.33	(7.18–7.40)	**0.024**
	Lactate, mmol/L (median, IQR)		5.2	(2.5–10.8)	3.3	(1.6–9.0)	3.8	(2.2–6.5)	3.3	(1.7–7.1)	0.430
	Sodium, mmol/L (median, IQR)		138	(135–141)	140	(135–143)	138	(137–140)	140	(137–142)	0.562
	Potassium, mmol/L (median, IQR)		4.5	(3.9–5.0)	4.3	(3.8–4.9)	4.2	(3.5–4.6)	4.1	(3.4–4.6)	0.262
	Creatinine, mg/dL (median, IQR)		1.6	(1.2–3.0)	1.6	(1.1–2.2)	1.2	(1.1–1.5)	1.4	(1.1–1.6)	0.154
	Hemoglobin, g/dL (median, IQR)		12.5	(10.6–13.9)	12.7	(10.3–14.0)	13.2	(12.0–14.6)	13.1	(10.3–14.4)	0.398
	White blood cell, 10^6^/mL (median, IQR)		16.9	(11.3–21.8)	15.4	(12.2–18.1)	16.6	(12.6–19.4)	17.0	(12.7–21.3)	0.676
	Platelets, 10^6^/mL (median, IQR)		213	(174–280)	238	(190–311)	231	(176–276)	220	(177–266)	0.762
	INR (mean, SEM)		1.6	(±0.1)	1.5	(±0.1)	1.4	(±0.1)	1.3	(±0.1)	0.471
	D-dimer, mg/l (mean, SEM)		23.3	(±2.9)	16.6	(±2.3)	16.2	(±2.8)	19	(±2.6)	0.197
	AST, U/L (mean, SEM)		1098	(±669)	474	(±155)	293	(±69)	327	(±72)	0.302
	ALT, U/L (mean, SEM)		450	(±228)	277	(±85)	158	(±29)	225	(±47)	0.361
	Bilirubin, mg/dL (mean, SEM)		0.7	(±0.1)	0.9	(±0.2)	0.9	(±0.1)	1.4	(±0.7)	0.486
	Troponin I, µg/L (mean, SEM)		75.7	(±42.9)	13.6	(±10.0)	18.7	(±10.4)	12.6	(±5.4)	0.120
	NT-pro BNP, pg/mL (median, IQR)		4311	(776–11,480)	6100	(1098–24,729)	2435	(168–9912)	491	(316–3118)	0.241
	Procalcitonin, ng/mL (mean, SEM)		2.4	(±1.5)	1.2	(±0.8)	0.2	(±0.1)	17.3	(±10.3)	0.135
	C-reactive protein, mg/L (mean, SEM)		18.5	(±4.6)	49.5	(±11.7)	25.7	(±8.6)	25.8	(±7.4)	0.065
Follow-up, 30 days											
	ICU time, days (median, IQR)		5	(1–11)	7	(2–10)	8	(3–13)	5	(3–12)	0.290

ALT, alanine aminotransferase; AST, aspartate aminotransferase; CS, cardiogenic shock; FiO_2_, fraction of inspired oxygen; LVEF, left ventricular ejection fraction; ICU, intensive care unit; INR, international normalized ratio; IQR, interquartile range; NT-pro BNP, amino terminal pro-B-type natriuretic peptide; PaO_2_, partial pressure of oxygen; PaCO_2_, partial pressure of carbon dioxide; PEEP, positive end-expiratory pressure; ROSC, return of spontaneous circulation; SEM, standard error of the mean; TAPSE, tricuspid annular plane systolic excursion; SCAI, Society for Cadiovascular Angiography and Interventions; ECMO, extracorporeal membrane oxygenation. Level of significance *p* < 0.05. Bold type indicates statistical significance.

Patients in the highest quartile for lung compliance demonstrated the least reliance on extracorporeal life support; notably, none of these patients required veno-arterial extracorporeal membrane oxygenation (ECMO) (17.1% vs. 13.9% vs. 28.6% vs. 0.0%; *p* = 0.009). Regarding respiratory parameters, patients with lung compliance below 23.9 mL/cmH_2_O exhibited significantly altered ventilatory settings. These patients had elevated PaCO_2_ (52 mmHg vs. 45 mmHg vs. 43 mmHg vs. 43 mmHg; *p* = 0.045) and were ventilated with higher PEEP (8 cmH_2_O vs. 9 cmH_2_O vs. 7 cmH_2_O vs. 7 cmH_2_O; *p* = 0.003), peak inspiratory pressure (31 cmH_2_O vs. 25 cmH_2_O vs. 20 cmH_2_O vs. 17 cmH_2_O; *p* = 0.001) and dynamic driving pressure (19 cmH_2_O vs. 16 cmH_2_O vs. 13 cmH_2_O vs. 11 cmH_2_O; *p* = 0.001). Conversely, the tidal volumes in this group were lower (371 mL vs. 451 mL vs. 458 mL vs. 580 mL; *p* = 0.001) compared to the other quartiles. Laboratory parameters showed an even distribution between the two groups, with the exception of pH levels, which varied significantly (7.21 vs. 7.25 vs. 7.27 vs. 7.33; *p* = 0.024) indicating a trend towards worsening acidosis with decreasing lung compliance.

**Fig. 2.**
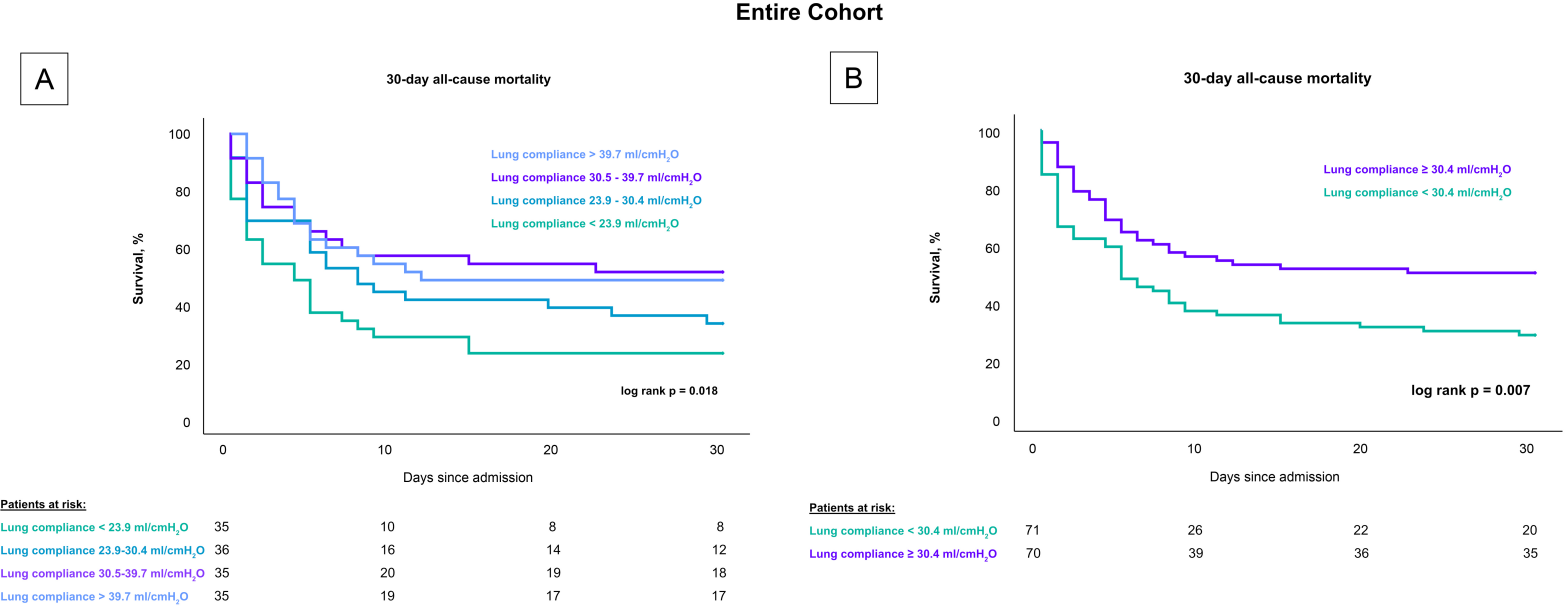
**Influence of lung compliance on 30-day all-cause mortality in CS-patients stratified by compliance levels**. Fig. 2 presents Kaplan-Meier survival curves for 30-day all-cause mortality in CS patients undergoing invasive mechanical ventilation. (A) displays mortality rates across quartiles of lung compliance, highlighting higher mortality rates in the lowest compliance quartile. (B) shows a comparison based on the median compliance level, with significantly higher mortality observed in patients below the median. These analyses underscore lung compliance as a predictive factor for mortality across all patients with CS, including those who experienced cardiac arrest. CS, cardiogenic shock.

**Fig. 3.**
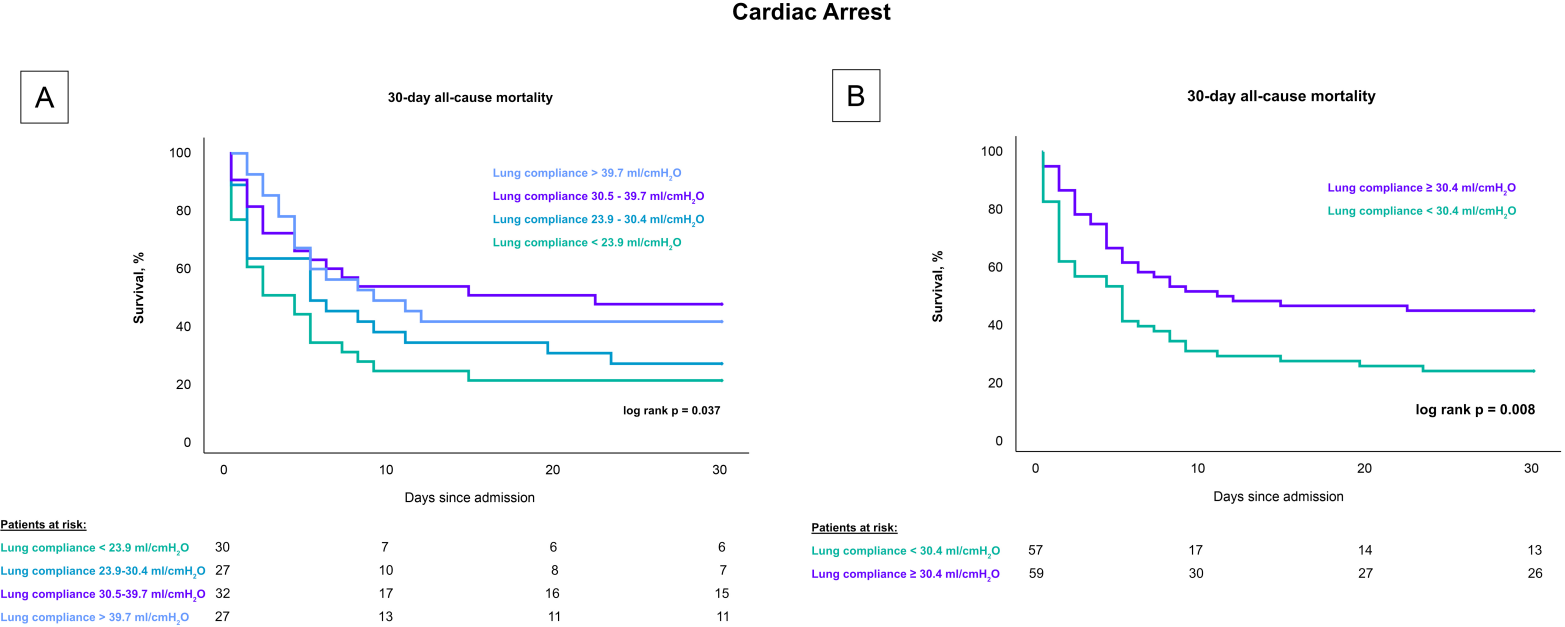
**Influence of lung compliance on 30-day all-cause mortality in CS-patients with cardiac arrest stratified by compliance levels**. Fig. 3 presents Kaplan-Meier survival curves for 30-day all-cause mortality among CS patients undergoing invasive mechanical ventilation who experienced a cardiac arrest. (A) depicts the mortality rates across four quartiles of lung compliance, demonstrating higher mortality in the lower compliance quartiles. (B) compares mortality rates between groups divided by the median lung compliance, showing significantly increased mortality in patients with compliance below the median. This figure emphasizes the prognostic value of lung compliance in critically ill cardiac patients. CS, cardiogenic shock.

Patients with lower lung compliance, when stratified by quartiles, had a greater 30-day risk of all-cause mortality compared to patients with higher compliance (log rank *p* = 0.018), as shown in Fig. 2. Specifically, the mortality rates were 77.1% for patients with lung compliance less than 23.9 mL/cmH_2_O, 66.7% for those with lung compliance between 23.9–30.4 mL/cmH_2_O, 48.6% for those between 30.5–39.7 mL/cmH_2_O, and 51.4% for those with lung compliance above 39.7 mL/cmH_2_O. After stratifying by the median, patients with lung compliance values below 30.4 mL/cmH_2_O demonstrated a higher mortality rate after 30 days compared to patients with lung compliance at or above 30.4 mL/cmH_2_O (50% vs. 71.8%; log rank *p* = 0.007). These findings remained consistent in a subset analysis that included only CS-patients with cardiac arrest, both when stratified by quartiles (80% vs. 74% vs. 53% vs. 59%; log-rank *p* = 0.037) and by the median (77.2% vs. 55.9%; log-rank *p* = 0.008), as shown in Fig. 3.

As detailed in Table 3, univariable Cox regression analysis demonstrated that lung compliance below 30.4 mL/cmH_2_O was significantly associated with the primary endpoint of 30-day all-cause mortality in the entire cohort (HR = 1.760; 95% CI = 1.143–2.708; *p* = 0.032), and in the subgroup of CS-patients with cardiac arrest (HR = 1.783; 95% CI = 1.134–2.804; *p* = 0.012). This association persisted even after multivariable adjustment, indicating that lower lung compliance under 30.4 mL/cmH_2_O remained a significant predictor of all-cause mortality in the entire cohort (HR = 1.698; 95% CI = 1.085–2.659; *p* = 0.021).

**Table 3.** **Factors influencing 30-day all-cause mortality (Cox regression analysis)**.

Variables	Entire cohort					
	Univariable			Multivariable^a^		
	HR	95% CI	*p* value	HR	95% CI	*p* value
Age	1.011	0.995–1.026	0.181	-	-	-
Body mass index (kg/m^2^)	0.999	0.958–1.042	0.962	-	-	-
Sex (male)	1.150	0.746–1.772	0.527	-	-	-
Lactate (mmol/L)	1.133	1.089–1.178	**0.001**	1.092	1.045–1.141	**0.001**
Norepinephrine (µg/kg/min)	1.306	1.126–1.514	**0.001**	-	-	-
Acute physiology score	1.086	1.039–1.134	**0.001**	1.051	1.001–1.104	**0.048**
SCAI CS stage	1.556	1.072–2.259	**0.020**	-	-	-
PaCO_2_ (mmHg)	0.995	0.978–1.012	0.563	-	-	-
PaO_2_/FiO_2_ ratio	1.000	0.998–1.001	0.774	-	-	-
Pneumonia	2.022	1.319–3.101	**0.001**	1.772	1.123–2.797	**0.014**
PEEP (cmH_2_O)	1.039	0.958–1.126	0.356	-	-	-
Peak inspiratory pressure (cmH_2_O)	1.029	0.998–1.062	0.064	-	-	-
CS cause	1.063	0.932–1.211	0.364	-	-	-
Troponin I (µg/L)	1.002	1.001–1.003	**0.002**	-	-	-
Lung compliance <30.4 mL/cmH_2_O	1.760	1.143–2.708	**0.032**	1.698	1.085–2.659	**0.021**
Variables	Cardiac arrest					
	Univariable			Multivariable^b^		
	HR	95% CI	*p* value	HR	95% CI	*p* value
Age	1.015	0.999–1.032	0.072	-	-	-
Body mass index (kg/m^2^)	1.025	0.971–1.081	0.374	-	-	-
Sex (male)	1.361	0.855–2.166	0.193	-	-	-
Lactate (mmol/L)	1.132	1.087–1.178	**0.001**	1.092	1.043–1.144	**0.001**
Norepinephrine (µg/kg/min)	1.248	1.063–1.466	**0.007**	-	-	-
Acute physiology score	1.099	1.046–1.155	**0.001**	1.058	1.002–1.118	**0.042**
PaCO_2_ (mmHg)	0.990	0.973–1.008	0.274	-	-	-
PaO_2_/FiO_2_ ratio	1.000	0.998–1.001	0.696	-	-	-
Pneumonia	2.185	1.389–3.437	**0.001**	1.824	1.125–2.959	**0.015**
PEEP (cmH_2_O)	1.032	0.948–1.124	0.463	-	-	-
Peak inspiratory pressure (cmH_2_O)	1.023	0.990–1.057	0.180	-	-	-
CS cause	1.113	0.976–1.269	0.111	-	-	-
Troponin I (µg/L)	1.002	1.001–1.003	**0.004**	-	-	-
Lung compliance <30.4 mL/cmH_2_O	1.783	1.134–2.804	**0.012**	1.523	0.952–2.438	0.080

CI, confidence interval; CS, cardiogenic shock; FiO_2_, fraction of inspired oxygen; HR, hazard ratio; PaCO_2_, partial pressure of carbon dioxide; PaO_2_, partial pressure of oxygen; PEEP, positive end expiratory pressure; SCAI, Society for Cardiovascular Angiography and Interventions. Level of significance *p* < 0.05. Bold type indicates statistical significance. ^a^ Variables in the multivariable regression model: Lactate, pneumonia, acute physiology score, SCAI CS stage, lung compliance <30.4 mL/cmH_2_O. ^b^ Variables in the multivariable regression model: Lactate, acute physiology score, pneumonia, lung compliance <30.4 mL/cmH_2_O.

Additionally, the multivariable Cox regression model revealed other significant predictors of the primary endpoint at 30 days. Lactate levels (HR = 1.092; 95% CI = 1.045–1.141; *p* = 0.001), acute physiology score (HR = 1.051; 95% CI = 1.001–1.104; *p* = 0.048), and the presence of pneumonia (HR = 1.772; 95% CI = 1.123–2.797; *p* = 0.014) were associated with increased mortality. However, in the subgroup analysis including only CS-patients with cardiac arrest, the association between lung compliance below 30.4 mL/cmH_2_O and all-cause mortality did not reach statistical significance (HR = 1.523; 95% CI = 0.952–2.438; *p* = 0.080). In contrast, lactate levels (HR = 1.092; 95% CI = 1.043–1.144; *p* = 0.001), acute physiology score (HR = 1.058; 95% CI = 1.002–1.118; *p* = 0.042) and existence of pneumonia (HR = 1.824; 95% CI = 1.125–2.959; *p* = 0.015) remained significant indicators of mortality risk in this specific patient group.

**Fig. 4.**
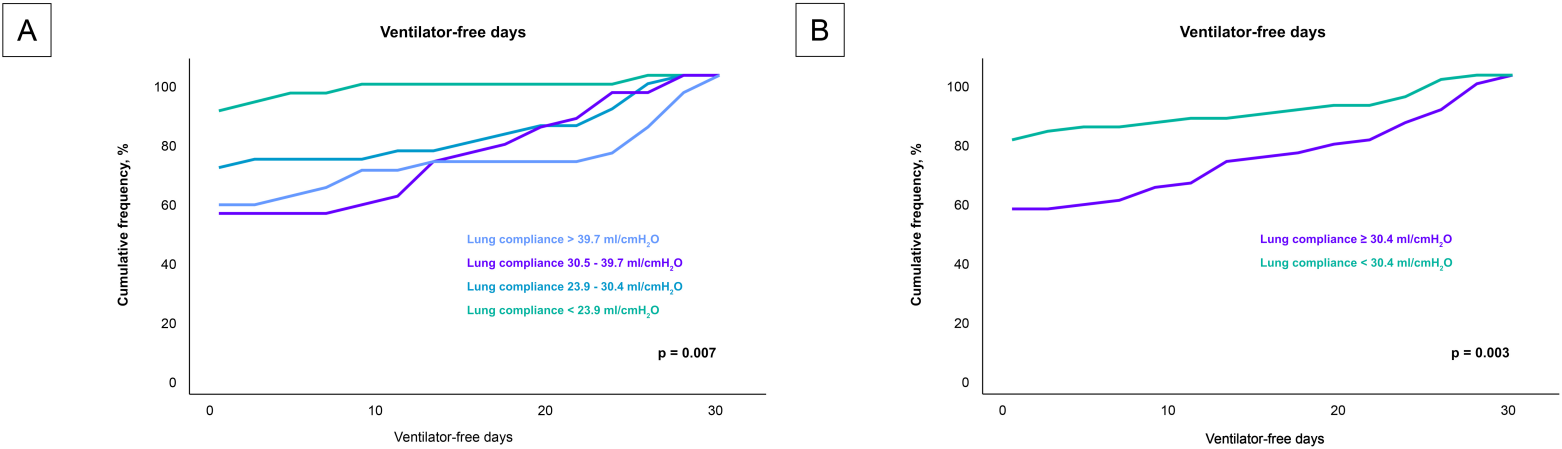
**Impact of lung compliance on ventilator-free days across compliance levels**. Fig. 4 details the relationship between lung compliance and the number of ventilator-free days among the entire cohort, analyzed in two different stratifications. (A) compares the ventilator-free days across quartiles of lung compliance, highlighting a decrease in ventilator-free days with lower lung compliance. (B) provides an analysis based on the median lung compliance value of 30.4 mL/cmH_2_O, further illustrating that patients below this median experience significantly fewer ventilator-free days. These visualizations emphasize how variations in lung compliance could influence the duration of mechanical ventilation required by patients, potentially impacting their recovery trajectory.

Analysis of the secondary endpoint as presented in Fig. 4, revealed a significant correlation between lung compliance and ventilator-free days. Patients with lower lung compliance experienced fewer ventilator-free days (*p* = 0.007). This trend was robust and remained statistically significant even when patients were stratified by the median lung compliance value of 30.4 mL/cmH_2_O (*p* = 0.003).

The correlation between lung compliance and laboratory and clinical data is shown in Table 4. Notably, there were moderate inverse correlations with sex (r = –0.222; *p* = 0.001), acute physiology score (r = –0.144; *p* = 0.014), PaCO_2_ (r = –0.159; *p* = 0.006). This inverse correlation also held true for mechanical ventilation settings including PEEP (r = –0.195; *p* = 0.002) and peak inspiratory pressure (r = –0.583; *p* = 0.001).

**Table 4.** **Associations between lung compliance with key laboratory and clinical parameters**.

	Lung compliance	
	r	*p* value
Age	–0.001	0.987
Body mass index (kg/m^2^)	–0.055	0.342
Sex (male)	–0.222	**0.001**
Lactate (mmol/L)	–0.103	0.074
Norepinephrine (µg/kg/min)	–0.042	0.486
Acute physiology score	–0.144	**0.014**
SCAI CS stage	–0.030	0.659
PaCO_2_ (mmHg)	–0.159	**0.006**
PaO_2_/FiO_2_ ratio	0.103	0.080
Pneumonia	–0.018	0.796
PEEP (cmH_2_O)	–0.195	**0.002**
Peak inspiratory pressure (cmH_2_O)	–0.583	**0.001**
Heart rate (bpm)	–0.043	0.457
Troponin I (µg/L)	0.026	0.666

Bpm, beats per minute; CS, cardiogenic shock; FiO_2_, fraction of inspired oxygen; ICU, intensive care unit; PaO_2_, partial pressure of oxygen; PaCO_2_, partial pressure of carbon dioxide; PEEP, positive end expiratory pressure; SCAI, Society for Cardiovascular Angiography and Interventions. Level of significance *p* < 0.05. Bold type indicates statistical significance.

## 4. Discussion

The primary objective of this study was to explore the prognostic influence of lung compliance at the time of ICU admission in patients with CS who required invasive mechanical ventilation. Our findings indicate that lower lung compliance is significantly associated with increased 30-day all-cause mortality. After stratifying by the median, we determined that patients with lung compliance below the median value of 30.4 mL/cmH_2_O experienced higher mortality rates than those with higher lung compliance. These findings persisted even after adjusting for multiple variables. Furthermore, patients with low lung compliance also had fewer ventilator-free days, suggesting a prolonged need for mechanical ventilation. These observations underscore the potential of lung compliance, a readily measurable parameter in ventilated CS patients, as a valuable tool for risk stratification.

Lung compliance is a critical parameter in the management of patients with ARDS and serves both diagnostic and prognostic purposes [4, 20, 21]. While it is well-established in ARDS, its implications in heart failure have predominantly been explored in spontaneously breathing patients. Studies in this group have consistently shown that lung compliance decreases with the manifestation of left ventricular failure regardless of the underlying NYHA class or pulmonary vascular pressure [5, 6, 7]. Observational data indicate a direct correlation between the degree of reduced lung compliance and higher pulmonary capillary wedge pressure [6]. In patients with CS, elevated pulmonary capillary wedge pressures are common, often leading to pulmonary edema and, consequently, reduced lung compliance [9, 22]. However, specific details on how varying degrees of reduced lung compliance affect CS patients remain sparse. Insights from a subanalysis of the LUNG SAFE study, focusing on patients with isolated cardiogenic pulmonary edema undergoing non-invasive or invasive ventilation, reveal that higher peak, plateau and driving pressures—markers of more severe disease—were associated with increased mortality [9]. This was accompanied by a reduction in lung compliance [9], which could explain the higher pressures needed for ventilation. Lung compliance in the high-pressure group was around 30 mL/cmH_2_O, correlating closely with results from the current study where a significant differentiation in Kaplan-Meier analysis was observed for lung compliance below 30.4 mL/cmH_2_O.

In the present study the majority of the included CS-patients suffered from cardiac arrest. Previous research has consistently demonstrated that lung compliance decreases after cardiac arrest from different causes, potentially exacerbating the outcome [10, 11, 12, 23]. In our analysis, CS patients with accompanied cardiac arrest demonstrated higher mortality rates when lung compliance was in the lower quartiles. This supports the hypothesis that post-cardiac arrest syndrome—characterized by complications such as chest compression, inflammation and aspiration pneumonia—could induce lung edema, thus impacting lung compliance and influencing study outcomes [24, 25]. However, when stratified by median lung compliance and adjusted for potential cofactors using multivariable Cox regression, the association of reduced lung compliance (<30.4 mL/cmH_2_O) with 30-day all-cause mortality did not reach the level of significance in the subgroup of patients with cardiac arrest. This suggests that while lung compliance is a critical factor in the context of post-cardiac arrest syndrome, its influence extends beyond this subgroup. Indeed, in the broader cohort of CS patients, including those without cardiac arrest, reduced lung compliance remained significantly associated with increased mortality even after controlling for multiple variables. This highlights the importance of lung compliance not only in the immediate aftermath of cardiac arrest but also as a general prognostic factor in cardiogenic shock management.

This study is the first to demonstrate that lung compliance may impact in risk prediction in patients with CS. This aligns with the growing body of evidence indicating that mechanical ventilation in patients with CS significantly impact mortality outcomes [26, 27]. Recent research, including a study by Povlsen *et al*. [26], found that non-survivors of CS due to acute myocardial infarction had notably higher ventilator pressure settings [26]. Our findings extend this observation, showing that patients with lower lung compliance not only require higher ventilatory pressure settings but also exhibit higher 30-day all-cause mortality.

The large retrospective cohort study by Povlsen *et al*. [26] showed that such correlations were predominantly observed in patients who had not experienced out-of-hospital cardiac arrest. This reinforces the notion that the effects of CS on patient outcomes extend beyond those typically seen in post-cardiac arrest syndrome. It suggests that the management strategies for invasively ventilated cardiac patients should consider the distinct impact of CS itself, independent of complications arising from cardiac arrest.

The underlying mechanisms contributing to the observed relationships in this study are likely multifactorial. First, the direct impact of positive pressure mechanical ventilation on lung tissue must be considered. Patients with CS who exhibit low lung compliance typically require higher positive pressure to ensure adequate ventilation. This necessity arises particularly because pulmonary capillary wedge pressure is generally increased in cases of left ventricular failure, commonly leading to pulmonary edema in these patients [9, 22]. With increasing lung fluid, ventilated lung volume decreases and lung compliance reduces, mirroring the “baby lung” concept observed in patients with ARDS [4, 28]. Consequently, areas of the lung filled with fluid need a higher pressure of mechanical ventilation, resulting in higher shear and lung stress, which is known to induce ventilator induced lung injury (VILI) [29, 30, 31, 32, 33, 34, 35]. This hypothesis is supported by our data where patients with lower lung compliance were initially ventilated with median driving pressure of 19 cmH_2_O, surpassing the threshold for lung-protective ventilation strategies [31]. Such high pressures can induce barotrauma, potentially leading to VILI, which can be prevented by applying low positive pressure ventilation [31]. Furthermore, adopting low pressure ventilation strategies might reduce the incidence of ARDS and pneumonia, further supporting the need for careful management of ventilatory pressures in this patient population [36].

In addition to the direct impacts on lung tissue, the need for higher pressure settings might also lead to an acceleration of systemic inflammation. Low pressure ventilation in ARDS patients was associated with lower levels of interleukin 6, which is an inflammatory marker [31]. This reduction in systemic inflammation could critically influence the progression of CS, as ongoing systemic inflammation exacerbates the shock condition and can accelerate patient deterioration [2, 37, 38]. Finally, the high-pressure ventilation caused by low lung compliance might have direct negative cardiovascular effects. An analysis by Rali *et al*. [27] of ventilator parameters in 2226 CS patients on extracorporeal life support suggested a cardioprotective effect of low-pressure ventilation. The study found that CS-patients managed with low pressure ventilation had the lowest mortality rates [27]. This observation is particularly significant as mortality in patients on extracorporeal life support is often not primarily due to respiratory failure. The authors suggest that low-pressure ventilation may provide cardio-protective benefits by influencing transmural pressures and the mechanical loading of the right and left ventricles. This hypothesis introduces an additional dimension to the benefits of low-pressure ventilation, suggesting that it could have systemic cardiovascular advantages beyond the respiratory system.

Taking these potential effects into account, we can hypothesize implications for daily clinical care. Patients with low lung compliance, who represent a high-risk subgroup within the CS population, require special attention. Pulmonary edema is a critical factor in the development of low lung compliance, suggesting that reducing lung fluid through diuretic therapy or renal replacement therapy may be beneficial [39]. Furthermore, low lung compliance may obstruct the application of low pressure ventilation. Therefore, in some patients, lung protective ventilation could be solely provided in prone positioning. However, it can be dangerous to place patients in prone position when they are experiencing CS since they are hemodynamically unstable. This challenge is reflected in our study’s real-world data, where a significant portion of the cohort suffered from cardiac arrest and was classified as SCAI shock stage E, resulting in high mortality rates. Consequently, prone positioning was only feasible for a small subset of patients. Despite these concerns, a study by Ruste *et al*. [40] has indicated that prone positioning was shown to increase cardiac index in patients with ARDS. Considering this, when the hemodynamic condition permit, it may be beneficial to place CS-patients in the prone position to improve survival. However, to corroborate this hypothesis, randomized controlled trials are needed.

This study contains a few limitations. Although we used multivariable Cox regression to account for potential cofounders, results may still be influenced by measurable or unmeasured cofounding factors due to the single-center and observational design of the study. Consequently, these findings should be considered as hypothesis generating rather than confirmatory because a direct causal association could not be demonstrated. While lung compliance might be a helpful risk predictor in CS, it should not be used in isolation. Rather, it should be integrated with additional parameter to enhance its predictive accuracy. Another limitation is the relatively small sample size of the study, which may have compromised its statistical power and influenced the outcomes. Therefore, the current findings require a reassessment as part of a more extensive investigation in larger trials. Additionally, due to the small sample size in the subgroup of patients without cardiac arrest on admission, survival analyses could not be performed, limiting the generalizability of our findings to the broader CS patient population without cardiac arrest. Additionally, the analysis did not include echocardiographic and invasive measurements which were inconsistent or only available in a small portion of the cohort. This included the measurement of the systolic pulmonary artery pressure, cardiac index, cardiac power output, or pulmonary capillary wedge pressure, before or after the implementation of invasive mechanical ventilation. Finally, Kaplan-Meier curves of the third and the fourth quartile of lung compliance showed crossing phenomena, which could be explained by specific clinical interventions and outcomes within these groups. Patients in the third quartile, who more frequently required extracorporeal life support and exhibited the highest rate of shock stage E, showed higher mortality in the initial days compared to the fourth quartile. Conversely, patients in the fourth quartile, who had higher applied tidal volume, may have succumbed to VILI, resulting in higher mortality after several days of ventilation. This suggests differential impacts on survival based on the management strategies and clinical conditions within these quartiles.

## 5. Conclusions

The present study demonstrated that patients with CS who exhibit low lung compliance experience a significantly higher 30-day all-cause mortality and fewer ventilator-free days. These findings provide new insights and call for more thorough research with prospective trials to better understand the impact of mechanical ventilation, including prone positioning, in patients with CS and cardiac arrest.

## Availability of Data and Materials

The datasets used and/or analyzed during the current study are available from the corresponding author on reasonable request.
